# Effect of gastric acid on the surface roughness and bacterial adhesion of bulk-fill composite resins

**DOI:** 10.1590/0103-6440202205140

**Published:** 2022-12-05

**Authors:** Deise Caren Somacal, Mariá Cortina Bellan, Marina Silveira Gregis Monteiro, Silvia Dias de Oliveira, Hélio Radke Bittencourt, Ana Maria Spohr

**Affiliations:** 1 Department of Restorative Dentistry, Pontifical Catholic University of Rio Grande do Sul, Porto Alegre, Rio Grande do Sul, Brazil; 2 Department of Immunology and Microbiology, Pontifical Catholic University of Rio Grande do Sul, Porto Alegre, Rio Grande do Sul, Brazil; 3 Department of Statistics, Pontifical Catholic University of Rio Grande do Sul, Porto Alegre, Rio Grande do Sul, Brazil

**Keywords:** bacterial adhesion, composite resins, gastric acid, surface roughness, tooth brusing

## Abstract

The purpose of this in vitro study was to evaluate the effect of gastric acid on the surface roughness and biofilm formation of bulk-fill composite resins. Twenty-seven samples of each composite resin were obtained: G1: Filtek Z250 XT (Z250), G2: Filtek Bulk Fill (FTK), G3: Tetric N-Ceram Bulk Fill (TTC), and G4: Aura Bulk Fill (AUR). The samples were quantitatively analyzed for surface roughness (Ra) using a roughness tester (n=15) and for biofilm formation (Cn) by the counting of colony-forming units (CFUs/mL) (n=9) in three different moments: after polishing (Ra0 and Cn0), after gastric acid immersion (Ra1 and Cn1), and after gastric acid and simulated tooth brushing (Ra2 and Cn2). Qualitative analysis through surface topography (n=3) was evaluated by scanning electron microscopy (SEM). Ra values were subjected to two-way repeated measures ANOVA, followed by Tukey’s test. Cn values were subjected to Kruskal-Wallis analysis, followed by multiple comparisons analysis (α=0.05). Z250 and FTK showed significant increases in surface roughness at Ra1. There were fewer CFUs/mL on TTC and AUR in relation to those of Z250 and FTK for Cn0, Cn1 and Cn2. The SEM images showed that gastric acid increased the formation of cracks, exposure of fillers and micro cavities for all composite resins. After tooth brushing, the topographical changes were more evident but did not influence biofilm formation. The gastric acid promoted both degradation of the surfaces and bacterial adhesion for all composite resins.

## Resumo

O objetivo deste estudo *in vitro* foi avaliar o efeito do ácido gástrico na rugosidade superficial e na formação do biofilme nas resinas compostas de incremento único. Vinte e sete amostras de cada resina composta foram confeccionadas: G1: Filtek Z250 XT (Z250), G2: Filtek Bulk Fill (FTK), G3: Tetric N-Ceram Bulk Fill (TTC), and G4: Aura Bulk Fill (AUR). As amostras foram analisadas quantitativamente quanto à rugosidade da superfície (Ra) usando um rugosímetro (n=15) e para formação de biofilme (Cn) pela contagem de unidades formadoras de colônias (UFC/mL) (n=9) em três diferentes momentos: após polimento (Ra0 and Cn0), após imersão em ácido gástrico (Ra1 and Cn1), e após ácido gástrico e simulação de escovação (Ra2 and Cn2). Análise qualitative da topografia superficial (n=3) foi avaliada por meio de microscopia eletrônica de varredura (MEV). Os valores de Ra foram analisados pela ANOVA de duas vias para amostras pareadas, seguido do teste de Tukey. Os valores de Cn foram submetidos ao teste de Kruskal-Wallis, seguido da análise de comparações múltiplas (α=0,05). Z250 e FTK tiveram aumento significativo na rugosidade superficial em Ra1. Houve menos CFUs/mL para TTC e AUR em relação à Z250 e FTK em Cn0, Cn1 and Cn2. As imagens em MEV mostraram que o ácido gástrico aumentou a formação de fendas, exposição das partículas e mcrocavidades para todas as resinas compostas. Após escovação, as mudanças topográficas foram mais evidentes, mas não influenciou na formação do biofilme. O ácido gástrico promoveu degradação da superfície e adesão bacteriana para todas as resinas compostas.

## Introduction

Dental erosion, which can be of extrinsic or intrinsic origin, is characterized by the loss of mineralized dental structure due to the frequent action of acids. Acid erosion of extrinsic origin comes from a diet rich in acidic foods and/or beverages, while that of intrinsic origin results from gastroesophageal reflux ^1^.

The gastric acid from the gastroesophageal reflux has a pH that ranges in value from 1 to 1.5, being more acidic than the critical pH for dental tissues, which is approximately 5.5 ^2^. Thus, gastric acid causes demineralization of enamel, dentin and cementum when in contact with the oral cavity ^2^.

Another disorder of intrinsic origin that generates acid erosion is bulimia nervosa. This disease is a behavioral eating disorder characterized by self-induced vomiting after uncontrolled food intake in order to avoid weight gain ^2^.

The regurgitated gastric acid in the oral cavity reaches the palatal and occlusal surfaces of the upper teeth and the occlusal surfaces of the lower teeth, and there may be variations of effect on the different dental areas. Common clinical features of intrinsic dental erosion are loss of tooth brightness, dentin exposure, tooth wear and restorative material changes ^1^ ^,^ ^3^.

Substances present in the oral environment, such as saliva and acids, reduce the physical and mechanical properties of restorative materials. Studies have shown that alteration of surface roughness occurs when composite resins are submerged in different acidic solutions and gastric acid ^4^ ^,^ ^5^ ^,^ ^6^.

Surface degradation facilitates the accumulation of bacterial plaque, and surface roughness greater than 0.2 μm favors the accumulation of bacterial plaque ^7^. An association between the surface roughness and bacterial adhesion was found to exist for resinous materials ^8^.

Wishing to facilitate practice in the clinical area resulted in bulk-fill composite resins. These materials allow the insertion of increments 4 to 10 mm in thickness without a prolonged polymerization time ^9^. Manufacturers have attempted to increase the depth of cure by a variety of methods including reducing the filler content, increasing filler particle size, and the use of additional photo-initiators ^9^.

There is a constant necessity to evaluate restorative materials under the challenging chemical conditions that occur in the oral cavity. One such challenging condition is the contact of gastric acid with resinous surfaces, which can cause chemical erosion. In addition, it is important to evaluate chemical erosion in association with the mechanical process of brushing, since it is a common daily oral hygiene habit. To the best of the author’s knowledge, there are currently no studies evaluating the effect of gastric acid on bulk-fill composite resins. Therefore, determining how gastric acid acts on the surfaces of bulk-fill composite resins is essential to proper clinical practice in patients with gastroesophageal reflux.

The aim of this study was to evaluate the effect of gastric acid on surface roughness and biofilm formation of three bulk-fill composite resins, in comparison with a conventional composite resin, with and without simulated tooth brushing. Complementary analysis using scanning electron microscopy (SEM) to evaluate surface topography was also performed. The study was conducted under the following null hypotheses: a) gastric acid does not significantly influence surface roughness and b) gastric acid does not significantly influence biofilm formation on composite resins.

## Materials and Methods

### Obtaining composite resin samples

Three bulk-fill and one conventional composite resins were used (Box 1). The composite resin samples were made using a silicone matrix with orifices of 5 mm in diameter and 4 mm in height. Twenty-seven samples were obtained for each composite resin: Group 1(control) Z250 XT (Z250), Group 2: Filtek Bulk Fill (FTK), Group 3: Tetric N-Ceram Bulk Fill (TTC), and Group 4: Aura Bulk Fill (AUR). The matrix was positioned on a glass plate and filled with composite resin. The Z250 resin composite was inserted in two increments of approximately 2 mm thickness. The FTK, TTC and AUR composite resins were each inserted in one increment of 4 mm thickness. A polyester strip was placed on each composite resin, followed by a glass plate, in order to obtain a flat surface. The composite resin increments were light-cured using the LED light unit Radii-cal (SDI, Vic., Australia) for 20 s at a distance of 1 mm from the sample surface. The light intensity was 1.000 mW/cm^2^ and controlled by a radiometer (Model 100 Demetron, Saint Louis, MN, USA).

**Box 1:**
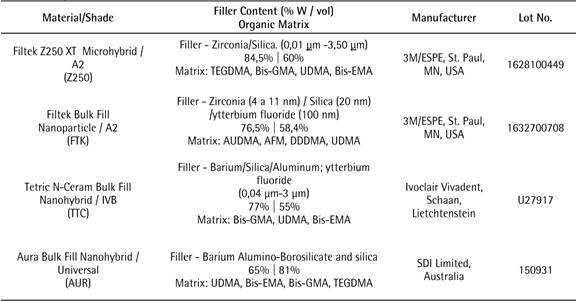
Composite resins used in the study.

Abbreviations: Bis-EMA, ethoxylated bisphenol-A glycidyl methacrylate; Bis-GMA, bisphenol-A glycidyl methacrylate; TEGDMA, triethyleneglycol dimethacrylate; UDMA, urethane dimethacrylate; AUDMA, aromatic urethane dimethacrylate; AFM, addition-fragmentation monomer; DDDMA, dodecanol dimethacrylate (DDMA).

The composite resin surface in contact with the polyester strip was finished with polishing discs (Sof-Lex Pop On, 3M/ESPE, St. Paul, MN, USA) of medium, fine and superfine grain. Each disc was applied for 15 s and by only one operator. After polishing, the samples were cleaned in ultrasound with distilled water for 10 min and then stored in distilled water at 37°C for 24 h.

### Surface roughness test

The initial surface roughness (Ra0) of the samples in each group (n=15) was measured with a roughness tester SL-201 (Mitutoyo Surftest Analyzer, Tokyo, Japan). Three consecutive measurements of the sample were taken in different regions (central, right and left), with a cutoff of 0.25. The mean values of roughness (Ra, in µm) were obtained for each sample.

### Immersion in gastric acid

The samples were immersed in gastric acid simulation solution for 24 h. The acid gastric solution was obtained by dissolving 2.0 g of sodium chloride and 3.2 g of pepsin in 7.0 mL of hydrochloric acid and water to obtain 1,000 mL. Each sample was immersed in 10 mL of gastric acid solution ^5^ ^,^ ^10^. After 24 h, the samples were washed with deionized water for 1 min. The surface roughness was measured again (Ra1).

### Simulated tooth brushing

A simulated tooth brushing machine, developed by the Idea Institute of the University, was used for this study. Each sample was fixed in the center (orifice) of an acrylic plate (55 x 25 x 4 mm), enabling the sample to remain 1 mm beyond the edge of the orifice that housed the sample. Utility wax was applied to fix the samples. Each plate was placed in an acrylic tank, which was attached to the brushing machine. The acrylic tank was filled with a mixture composed of 1 g of toothpaste (Colgate Total 12, Colgate-Palmolive, São Bernardo do Campo, SP, Brazil) per 1 mL of distilled water. Soft bristle Classic Colgate toothbrushes (Colgate-Palmolive, São Bernardo do Campo, SP, Brazil) were used, and a load of 200 g was applied. The speed of brushing was 250 cycles per minute, carried out in 10,000 cycles of simulated tooth brushing. The toothbrushes were changed every four brushed samples from each group. After the brushing cycle, the samples were washed in running water and ultrasonically cleaned in distilled water for 10 min, followed by drying with compressed air. The roughness of the surface was measured again (Ra2). The surface roughness reading was perpendicular to the brushing direction of the toothbrush bristles. For the correct positioning of the sample in the brushing machine and to always ensure readability in the same direction (perpendicular to the brushing), a mark with a diamond bur and high-speed hand piece was made on the border of each sample.

### Surface topography analysis by scanning electron microscopy (SEM)

Three samples (n=3) from each composite resin were used. Of the three samples, one was analyzed by SEM after polishing, the second after immersion in gastric acid, and the third after immersion in gastric acid and simulated tooth brushing. The samples were dried in a dehumidifier with silica gel for 72 h and fixed in the sampler with double-sided carbon adhesive tape (SPI, West Chester, PA, USA). The top surface was metallized with gold (Balzers, Liechtenstein) and observed by SEM (JSM 6060, Eindhoven, Netherlands) under 20,000X magnification.

### Biofilm analysis

The samples of composite resin were sterilized with ethylene oxide gas (ETR Sterilizer, Porto Alegre, RS, Brazil). The colony forming units (CFU/mL) were evaluated in triplicate for each group (n=9) after the following treatments: polishing (Cn0), gastric acid (Cn1), gastric acid and simulated tooth brushing (Cn2).

Streptococcus mutans (*S. mutans*) ATCC 25175, stored at -20°C, was obtained and cultivated in BHI (brain-heart infusion) for 24 h at 37°C in a bacteriological incubator. An aliquot of this primary culture was inoculated into a fresh BHI broth and incubated for an additional 24 h at 37°C. Subsequently, 100 μL of this culture, containing approximately 10^6^CFU/mL was used to inoculate 1 mL of BHI broth supplemented with 1% sucrose. The composite resin sample was then placed in the resulting culture and allowed to incubate for 24 h under microaerophilic conditions at 37°C. The samples were carefully removed from the culture and washed in 1 mL of 0.85% saline solution, removing the planktonic cells and keeping only the cells adhered to the surface of the samples; this process was performed twice.

*CFU/mL -* After the washes, the samples were placed in the ultrasonic bath for 10 min in order to disintegrate the biofilm. Subsequently, the saline solution containing the disaggregated cells was diluted to 10^-6^. The first three dilutions were performed using the gout technique and the last three using the scattering technique; both dilutions were performed on BHI agar, in triplicate. These two techniques were used according to the expected cellular concentrations at each dilution. The BHI agar plates were incubated in an oven at 37°C for 24 h, followed by CFU/mL count.

### Statistical analysis

Kolmogorov-Smirnov was performed to test normality of the data. After confirming the normality of surface roughness (p>0.05), two-way repeated measures analysis of variance (ANOVA) (material x treatment) was used, followed by Tukey’s test. As there was no normality for the CFU/mL count (p<0.05) and the homogeneity of variance assumption was violated (p<005), this variable was analyzed by Kruskal-Wallis nonparametric test, followed by multiple comparisons. The significance level was 5%. The software used was SPSS 10.0 (SPSS Inc., Chicago, IL, USA).

## Results

### Surface Roughness

According to the two-way ANOVA, the material factor (p=0.256) was not significant, while the treatment factor (p=0.01) and the interaction between the factors (p=0.013) were significant.

The four composite resins did not differ significantly from each other at Ra0 and Ra2. Z250 and FTK each had significant increases in surface roughness at Ra1 and significant decreases in surface roughness at Ra2 compared to that at Ra1. There were gradual increases in surface roughness at Ra0, Ra1 and Ra2 for TTC and AUR, but the differences were not significant (Table 1).

**Table 1:** Surface roughness means (μm) and standard-deviations (SD) of the composite resins.

Material	Ra0	Ra1	Ra2
	Surface roughness after polishing (µm) and SD	Surface roughness (µm) after gastric acid and SD	Surface roughness (µm) after gastric acid followed by tooth brushing and SD
Z250	0.18ª^B^ (0.08)	0.27ª^bA^ (0.14)	0.21ª^B^ (0.08)
FTK	0.14ª^B^ (0.08)	0.31ª^A^ (0.22)	0.18ª^B^ (0.09)
TTC	0.13ª^A^ (0.04)	0.16^bA^ (0.11)	0.23ª^A^ (0.20)
AUR	0.15ª^A^ (0.06)	0.16^bA^ (0.08)	0.22ª^A^ (0.07)

Means followed by different lowercase letters in columns and different capital letters in lines represent significant differences according to Tukey’s test (α=0.05).

### Surface Topography Analysis by SEM

**Figure 1:**
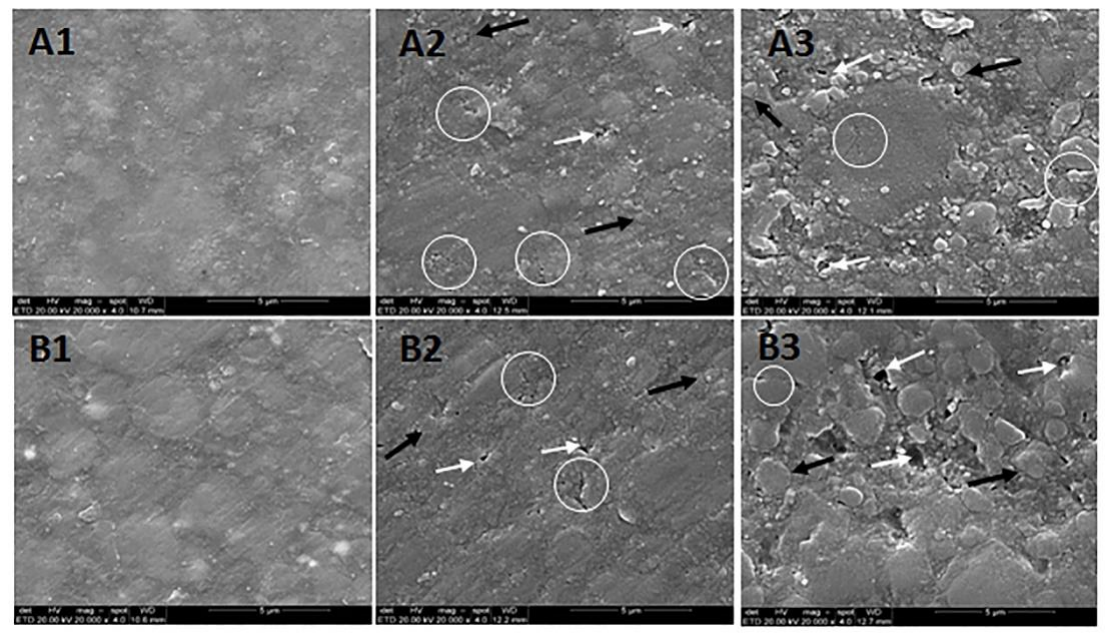
SEM images (20,000x) of the surface topography of Filtek Z250 XT and Filtek Bulk Fill. (A) Filtek Z250 XT and (B) Filtek Bulk Fill after polishing ^1^, after gastric acid ^2^, after gastric acid and simulated tooth brushing ^3^. Circles: cracks; Black arrows: fillers exposure; White arrows: microcavities.

**Figure 2:**
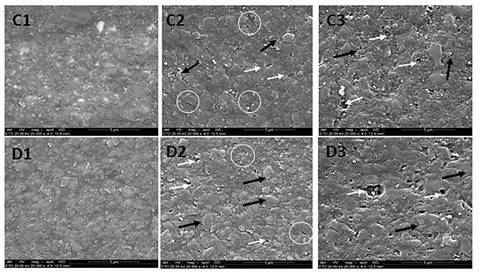
SEM images (20,000x) of the surface topography of Tetric N-Ceram Bulk Fill and Aura Bulk Fill. (C) Tetric N-Ceram Bulk Fill and (D) Aura Bulk Fill after polishing ^1^, after gastric acid ^2^, after gastric acid and simulated tooth brushing ^3^. Circles: cracks; Black arrows: fillers exposure; White arrows: microcavities.

The polished surfaces of the composite resins Z250, FTK, TTC and AUR are shown in Figure 1 (A1 and B1) and Figure 2 (C1 and D1), respectively. After immersion in gastric acid, surface cracks (circles), exposure of the fillers (black arrows) and formation of microcavities (white arrows) occurred in Z250 (Figure 1A2). The same occurred in FTK (Figure 1B2), TTC (Figure 2C2) and AUR (Figure 2D2), but in different degrees of degradation. After immersion in gastric acid followed by simulated tooth brushing, greater degradation of the organic matrix occurred in all composite resins. There was crack formation, filler exposure and microcavity formation in Z250 (Figure 1A3) and FTK (Figure 1B3). TTC (Figure 2C3) and AUR (Figure 2D3) also showed greater degradation of the organic matrix, having filler exposure and microcavity formation.

### CFU/mL

According to the Kruskal-Wallis analysis, followed by multiple comparisons, there was a significant difference in CFU/mL count among the composite resins. At Cn0, Cn1 and Cn2 there were lower CFU/mL counts for TTC and AUR in relation to those for Z250 and FTK. However, these numerical differences were not always statistically different (Table 2). At Cn0, FTK obtained the highest CFU/mL count, differing significantly from that of TTC, which obtained the lowest CFU/mL count. At Cn1, there was an increase in the CFU/mL count for all composite resins, with significant increases occurring only for FTK. At Cn2, the CFU/mL counts reduced significantly for FTK and AUR in comparison to those observed at Cn1 (Table 2).

**Table 2:** Median values of CFU/mL10^6^ of the composite resins in the different treatments.

Material	Cn0	Cn1	Cn2
	After polishing (CFU/mL log^6^)	After gastric acid (CFU/mL log^6^)	After gastric acid and tooth brushing (CFU/mL log^6^)
Z250	7.00^abB^	16.00^abAB^	26.00^aA^
FTK	31.60^aB^	97.00^aA^	9.60^aB^
TTC	0.60^bA^	1.60^cA^	2.40ª^bA^
AUR	2.00^abAB^	3.60^bcA^	0.33^bB^

Medians followed by the same lowercase letter in the columns and uppercase in the lines do not differ significantly by the multiple comparison test.

## Discussion

In the present study, surface roughness (Ra) and CFU/mL (Cn) of three bulk-fill composites were evaluated in comparison to those of a conventional composite resin after polishing (Ra0, Cn0), immersion in gastric acid (Ra1, Cn1), and immersion in gastric acid followed by simulated tooth brushing (Ra2, Cn2). According to the results, gastric acid influenced the surface roughness of two composite resins. Therefore, the first hypothesis of the study was rejected.

Immersion in gastric acid (Ra1) significantly increased the surface roughness of Z250 and FTK. However, gastric acid did not influence surface roughness for TTC and AUR. Two factors may be related to the increase in surface roughness. The first factor may be degradation of the organic matrix by contact with the water of the gastric acid solution; the water penetrates the polymer chain, chemically degrades the polymer and leads to the creation of oligomers and monomers. The progressive degradation of this matrix leads to the formation of pores, through which the oligomers and monomers are released. The second factor may be degradation of the organic matrix by the acid pH of the solution; catalization of the ester groups of the dimethacrylates occurs, favoring the hydrolysis of these groups and formation of molecules of carboxylic acid and alcohol, which accelerate the degradation of the composite resin ^11^. Although these dimethacrylate monomers are present in the composition of the four investigated composite resins, the results suggest that Z250 and FTK experience greater organic matrix degradation in relation to that of TTC and AUR.

Z250 contains the TEGDMA monomer, which reduces the viscosity of the material while increasing its water absorption ^12^. Degradation of the composite resins by hydrolysis is directly related to the polymerization of the material, as well as to the monomeric composition. TEGDMA was shown to be more susceptible to hydrolysis than Bis-GMA and Bis-EMA, leading to increased material wear and surface roughness ^13^. AUR also contains TEGDMA; however, it did not show significant surface roughness alteration after immersion in gastric acid, which suggests differences in the percentages of this monomer in the compositions of the composite resins ^13^. FTK does not contain TEGDMA in its composition, and it also showed surface roughness alteration after immersion in acid gastric; a possible reason for this alteration is degradation of the silane bonding agent. FTK is a nanofiller composite resin and has a larger surface area between fillers than the other composite resins do, which allows more water to accumulate at the interfaces of the organic matrix, increasing water absorption and degradation ^13^.

In the present study, the association between gastric acid and simulated tooth brushing (Ra2) with toothpaste was also evaluated, since tooth brushing is part of common daily oral hygiene. Simulated tooth brushing abrasion is a methodology established in the literature; it is an important *in vitro* wear factor which simulates clinical conditions. A patient performs approximately 15 cycles for each session of brushing. Thus, maintaining an oral hygiene routine consisting of two daily brushing sessions, 10,000 cycles are performed by the end of one year, which is also the number of cycles applied to the samples ^14^. After simulated tooth brushing, there was no significant difference in surface roughness among the composite resins, nor a significant difference in comparison with Ra0. This result demonstrates that simulated tooth brushing, after immersion in gastric acid, causes similar alteration in the surface roughness of the composite resins. Other studies also did not observe differences in surface roughness of composite resins after subjection to 10,000 cycles of simulated tooth brushing with Colgate Total 12 toothpaste ^14^ ^,^ ^15^.

Although there was no significant difference in surface roughness between Ra0 and Ra1 for TTC and AUR, SEM images showed changes in the surface topographies of these composite resins as well as in those of Z250 and FTK. After immersion in gastric acid, cracks, filler exposure and microcavities occurred in all composite resins. These findings may be related to degradation of the organic matrix by the action of gastric acid, as well as by the absorption of water contained in the acid solution ^4^. Water absorption depends on the composition of the organic matrix and the quality of the bond between the organic matrix and the fillers ^13^. The cracks come from the increase in osmotic pressure at the organic matrix/filler interface and the consequent hydrolytic degradation of the material ^16^. The absorption of water can lead to the expansion and leaching of the monomers, favoring the formation of cracks ^17^. The cracks visualized in the present study were also detected by another study ^16^. Moreover, water absorption can generate hydrolytic degradation of the silane, favoring the detachment of the fillers from the organic matrix and, consequently, the formation of microcavities ^17^.

Although there were no significant differences in surface roughness between Ra1 and Ra2, SEM images demonstrated that the association of gastric acid with simulated tooth brushing caused a greater change in the surface topography of the composite resins, and the formation of cracks, exposure of fillers and microcavities. The simulated tooth brushing with toothpaste causes wear on the composite resin surface by abrasion process ^14^, and several mechanisms are related to the wear of composite resins: ^1^ organic matrix wear; ^2^ loss of fillers because of bond failure with the organic matrix; ^3^ loss of fillers due to shear of exposure fillers; ^4^ loss of fillers as a result of cracks in the organic matrix; and ^5^ air bubbles exposure intrinsic to the restorative process ^18^. The formation of microcavities was more evident after tooth brushing, which can be explained by the abrasive effect of the toothpaste and the bristles of the toothbrush, favoring the greater removal of the exposed fillers.

One of the important aspects of the surface roughness study is related to the bacterial adhesion and retention. In Ra0, all composite resins obtained surface roughness less than 0.2 μm. After gastric acid (Ra1) and after simulated toothbrushing (Ra2), the surface roughness of the composite resins was approximately at the stated threshold surface roughness of 0.2 µm, which can be considered a positive result since surface roughness above 0,2 μm favors an increase in bacterial colonization on the material surfaces ^7^.

The cracks and microcavities observed in SEM images are present in the matrix/filler interface and offer the possibility of bacteria adhesion by surface free energy ^19^. The accumulation of biofilm on the composite resin surface facilitates the degradation of the material by the low pH of the biofilm ^4^, in addition to favoring the formation of secondary caries, which is one of the factors responsible for the failure of composite resin restorations ^20^.

In relation to CFU/mL, gastric acid influenced the amount of bacteria that were adhered to the surface of the composite resins. Therefore, the second hypothesis was rejected. Preferably, a composite resin restoration should have low susceptibility to bacterial adhesion. Physically, bacterial adhesion and retention occur in four phases: 1) bacterium transport towards the surface, 2) initial bacterial adhesion, 3) attachment by specific interactions, and 4) surface colonization ^21^. The second and third stages are physico-chemically possible because a surface and a bacterium distantly interact among each other (approximately 50 nm) through a combination of van der Waal’s attractive forces and electrostatic repulsive forces. On rougher surfaces, bacteria are more protected against shear forces and are able to have enough time to bridge the distance or to reach direct contact. Thus, surface free energy and surface roughness are important factors which influence bacterial adhesion ^7^.

After application of gastric acid (Cn1), there was a higher CFU/mL for Z250 and FTK, and a lower CFU/mL for TTC and AUR. The higher CFU/mL for Z250 and FTK can be explained by the acidic pH of the solution that promotes surface changes, increasing surface roughness and the free surface energy, making the composite resin surface favorable for bacterial adhesion ^22^. The surface roughness of Z50 and FTK were higher after gastric acid and justify the higher CFU/mL obtained for these two composite resins in comparison with TTC and AUR. After simulated toothbrushing (Cn2), the CFU/mL decreased significantly for FTK and AUR; for Z250 and TTC, there was an increase in CFU/mL, but without a significant difference in comparison with that for Cn1. Therefore, simulated toothbrushing, in spite of showing a greater alteration of the surface topography of the composite resins, did not favor a significant biofilm formation. A possible explanation for this finding may be related to the use of a toothpaste that contains sodium fluoride.

Sodium fluoride in small concentrations exerts subtle antimicrobial action, presenting direct and indirect effects on *S. mutans*. In the direct effect, sodium fluoride prevents the increase in the number of *S. mutans* through the inhibition of critical metabolic processes In the indirect effect, sodium fluoride reduces environmental acidification in the biofilm ^23^.

Another component of the toothpaste is triclosan at 0.3% which may have influenced in the bacterial adhesion, since it has a broad spectrum of antimicrobial action. At low concentrations (0.2 to 0.5%), triclosan affects the metabolism of some bacterial species, such as *S. mutans*. This chemical substance prevents the synthesis of proteins when in bacteriostatic concentrations, disorganizing the cytoplasmic membrane of the bacteria and extravasating its intracellular contents ^24^, which may have contributed to the lower bacterial adhesion in the composite resin surfaces.

One of the limitations of the present study is the difficulty of simulating in laboratory the continuous effect of gastric acid coming from gastroesophageal reflux and the effect of saliva on the whole degradation process of the materials. Aiming at standardization, the composite resin samples were exposed to gastric acid for 24 h, and there was no contact with artificial saliva. Thus, this exposure to gastric acid characterized the worst scenario of acid erosion cases ^5^, with similar exposure to eight years in the mouth ^25^. In addition, it is an *in vitro* study, and care must be taken to extrapolate the results to clinical reality. However, the results suggest that bulk-fill composite resins may be affected by the chemical composition of gastric acid resulting from gastroesophageal reflux, degrading the surface of the material and promoting bacterial adhesion. As a continuation of this research line, it would be interesting to perform observational studies in patients with gastroesophageal reflux in order to analyze *in vivo* the changes that gastric acid can cause in composite resin restorations.

Despite the limitations of this *in vitro* study, it can be concluded that gastric acid promoted higher surface roughness and higher bacterial counts in Z250 and FTK than that in the TTC and AUR composite resins. In addition, gastric acid promoted alterations of the surface topographies of all composite resins, resulting in cracks, filler exposures and microcavities as well as greater bacterial adhesion. These topographic changes were more evident after simulated tooth brushing but did not cause a significant increase in the bacterial adhesion.
